# Case Report: Primary Ewing Sarcoma of the Penis With Multiple Metastases

**DOI:** 10.3389/fped.2020.591257

**Published:** 2021-01-07

**Authors:** Chuanxi Zheng, Yong Zhou, Yi Luo, Hongying Zhang, Chongqi Tu, Li Min

**Affiliations:** ^1^Department of Orthopedics, West China Hospital, Sichuan University, Chengdu, China; ^2^Department of Pathology, West China Hospital, Sichuan University, Chengdu, China

**Keywords:** Ewing sarcoma, extra-skeletal Ewing sarcoma, penis, metastasis, diagnosis

## Abstract

**Background:** Ewing sarcoma is the second most common malignant bone tumor in children, but it rarely originates from extra-skeletal sites. The commonly involved sites of soft tissue include paravertebral spaces, lower extremities, the pelvis, head, and neck, while primary extra-skeletal Ewing sarcoma (EES) located in the genitals is extremely rare.

**Case Presentation:** We report a young patient who presented to our hospital with a painful erection of the penis and limited motion of the left hip. Magnetic resonance imaging showed a hyperintense mass with invasion of adjacent tissue in the penis and a heterogeneously high signal lesion in the left proximal femur. ^18^F-fluorodeoxyglucose positron-emission tomography detected widespread metastatic lesions in the bilateral lung and multiple skeletons. An incisional biopsy of the penis was performed; the histopathological findings and EWS gene translocation identified by molecular analysis confirmed the diagnosis of Ewing sarcoma. Subsequently, the punch-biopsy specimen from the left femur showed undifferentiated small round cells, a finding consistent with the microscopic presence of Ewing sarcoma metastasis. However, after the first course of multiagent chemotherapy, the penile mass did not obtain stabilization but instead grew progressively with surface ulceration and multidrug resistant bacteria infection. Despite receiving antibiotics and maximal supportive therapy, the patient died from sepsis and lung metastasis complications in the intensive care unit 2 months later.

**Conclusion:** This case indicates that although EES as a subtype of Ewing sarcoma is rare, it can occur virtually in any soft tissue site, even in the genitals. Therefore, clinicians need to distinguish this entity from other soft tissue sarcomas with rapid progression since early diagnosis and timely treatment of EES are pivotal for a favorable prognosis.

## Introduction

Ewing sarcoma belongs to a larger subset known as the Ewing sarcoma family of tumors, including Ewing sarcoma, peripheral primitive neuroectodermal tumors of bone or soft tissues, and Askin tumors (1). Ewing sarcoma is the second most common malignant bone tumor in children, characterized by poorly differentiated, aggressive clinical features with a high rate of local recurrence and distant metastasis (1). Classically, the lesions most commonly involve flat bones of the axial skeleton and the diaphysis of long bones, while 10–20% of cases originate from soft tissue sites (2). Extra-skeletal Ewing sarcoma (EES) is frequently located in the extremities, chest wall, paravertebral space, and retroperitoneum, but some rare anatomic locations have also been reported, such as the jejunum, heart, larynx, vulva, kidneys (3–7). To our knowledge, the occurrence of EES in the penis is extremely rare, and there are only eight relevant case reports that have been discussed (8, 9). However, none of those reported cases was accompanied by multiple metastatic lesions, especially osseous metastases. Here, we present a rare case of EES in the penis with multiple metastases in the bones and lungs.

## Case Presentation

**Figure 1:**
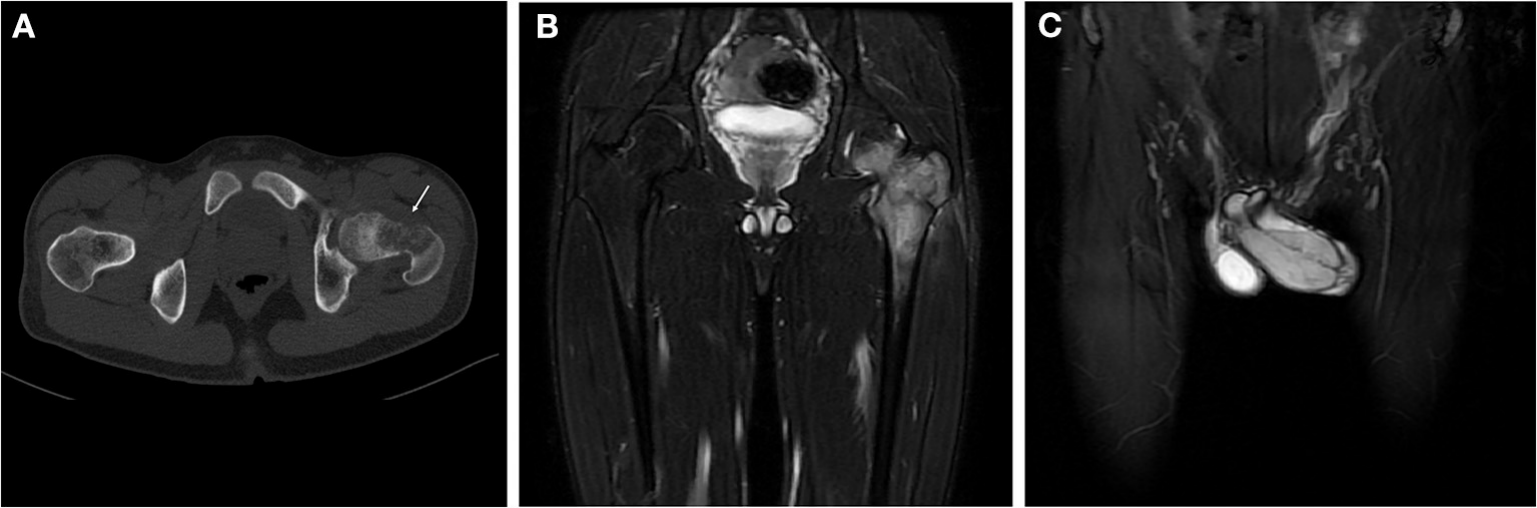
Radiological images of the pelvis. Axial CT (**A**, bone window) showed an aggressive lytic lesion involving the left femoral neck and trochanteric region associated with cortical destruction and limited periosteal reaction. A coronal fat-saturated T2-weighted MRI image of the pelvis **(B)** demonstrated a heterogeneously high signal lesion in the left proximal femur that was limited in the medullary cavity without extraosseous extension. Meanwhile, MRI images **(C)** showed a hyperintense soft tissue mass in the dorsal side of the penis with poorly defined zones of high signal in the *corpus cavernosum* around the tumor.

**Figure 2:**
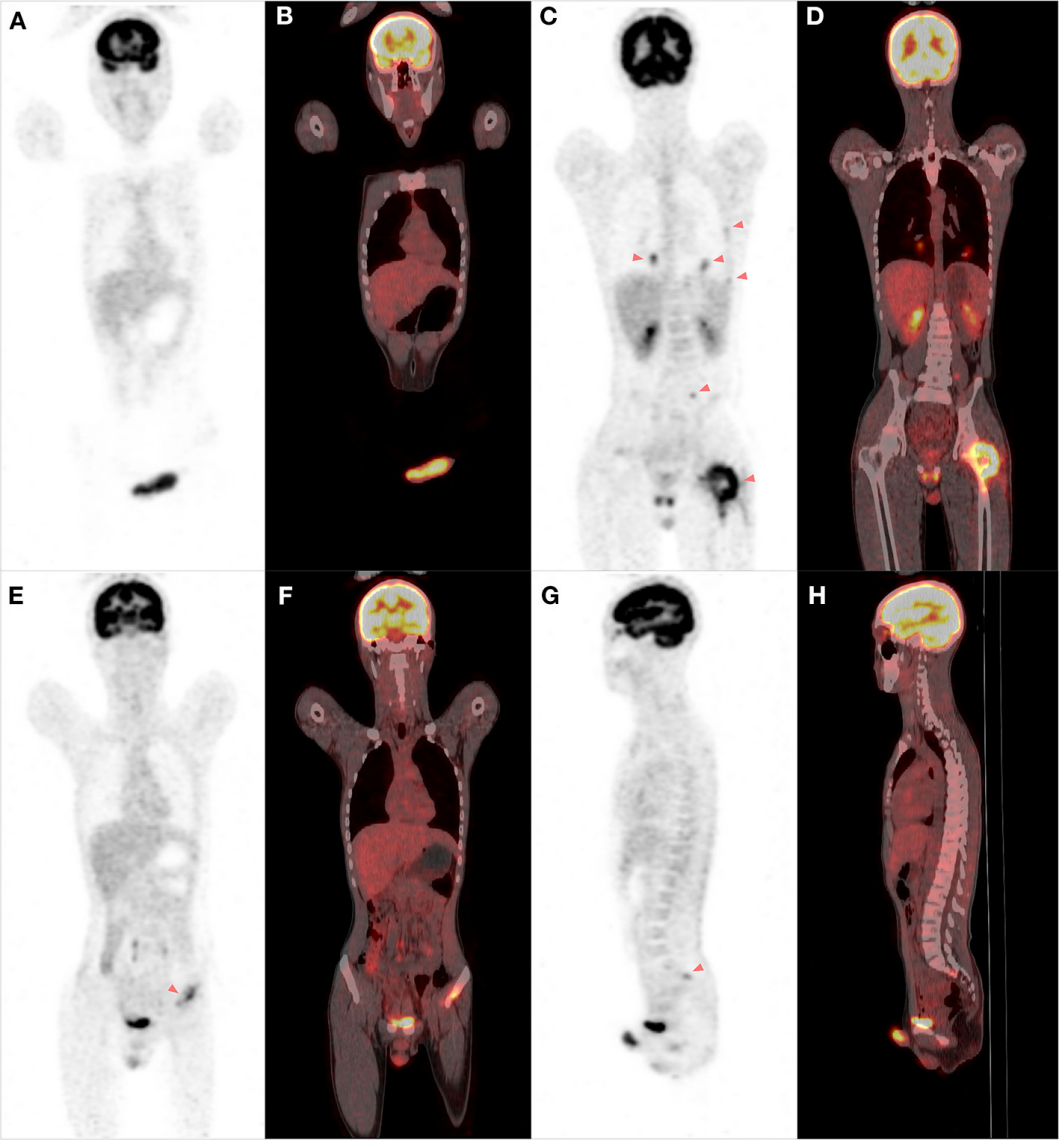
The FDG PET/CT scan of the whole body. A PET-CT scan showed increased fluorodeoxyglucose uptake in the lesions of the penis and *corpus cavernosum* **(A,B)**. Multiple lesions with uptake were detected in the bilateral lungs, ribs, left proximal femur, lumbar vertebrae, and the left acetabulum and sacrum, indicating the possibility of metastatic disease **(C–H)**.

**Figure 3:**
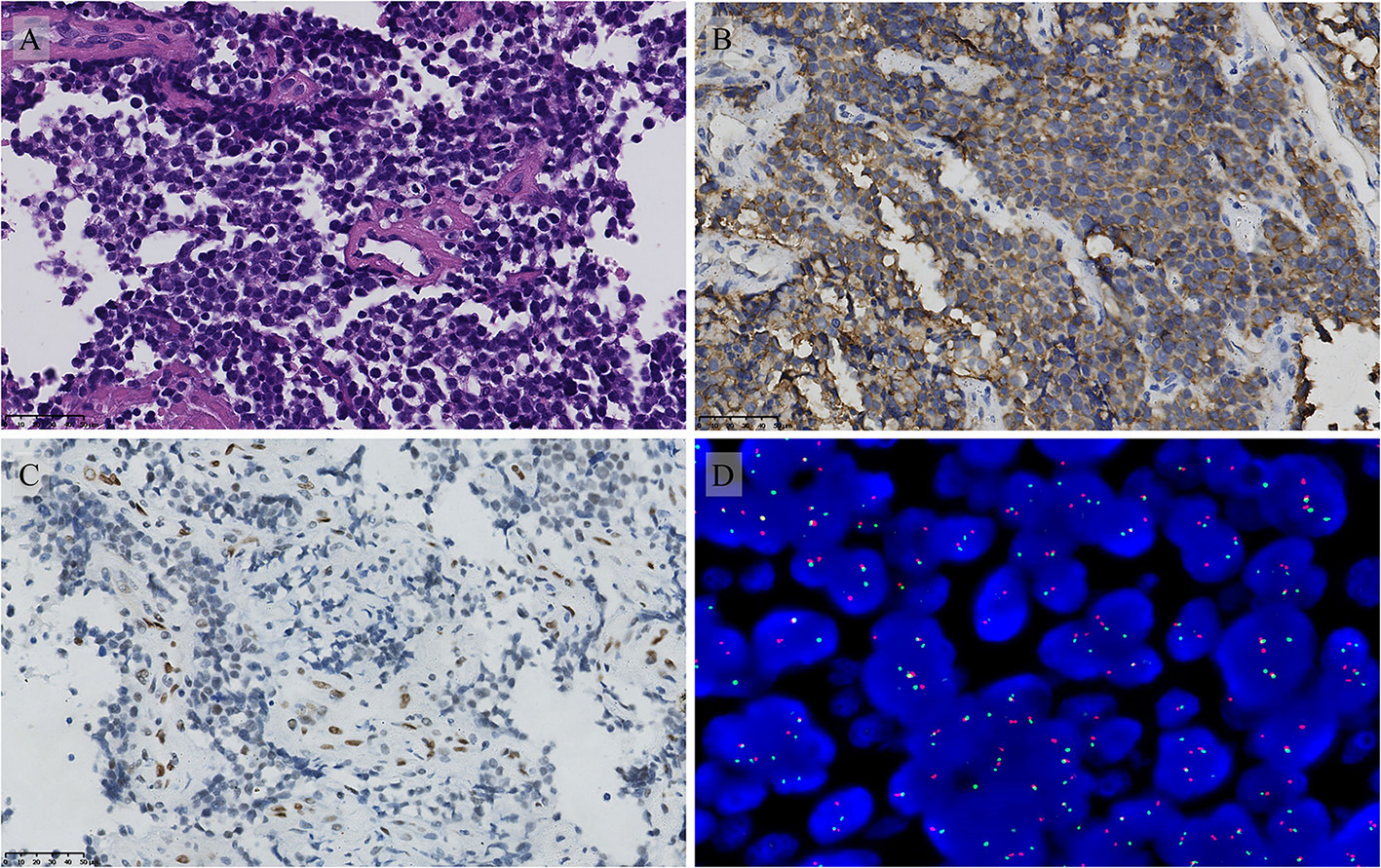
Pathological features of the penile lesion. **(A)** The H and E stain showed that the tumor is composed of uniform small round cells (magnification 400 ×). The immunohistochemical stain showed **(B)** Diffuse membranous CD99 positivity and focal FLI1 positivity **(C)** (magnification 400 ×). FISH molecular analysis demonstrated the EWS gene translocation **(D)**.

A 17-year-old young patient presented to our orthopedic clinic with a 1-week history of limited motion of the left hip and a painful erection of the penis for 3 weeks. The local hospital initially suspected priapitis and administered a treatment of non-specific antibiotics, but the pain was not relieved. Physical examination revealed tenderness in the left inguinal trochanteric region and limited mobility of left hip; a firm, palpable, tender mass was detected at the dorsal region of the penis. Computed tomography showed an aggressive lytic lesion involving the left femoral neck and trochanteric region with cortical destruction and limited periosteal reaction (Figure 1). Magnetic resonance imaging (MRI) of the pelvis displayed a heterogeneously high-signal lesion without observable extraosseous extension in the left proximal femur. Meanwhile, MRI images demonstrated a hyperintense lesion measuring approximately 5.1 cm × 2.6 cm × 2.0 cm on the dorsal side of the penis, with poorly defined zones of high signal in the corpus cavernosum around the tumor (Figure 1). Bone scintigraphy presented the diffusely increased activity in the left proximal femur. An ^18^f-fluorodeoxyglucose positron-emission tomography scan was performed to evaluate other sites' involvement, which showed increased fluorodeoxyglucose uptake in the lesions of the penis and left proximal femur with maximum SUV values of 6.7 and 8.2, respectively (Figure 2); multiple lesions with mild uptake were also found in the bilateral lungs, ribs, lumbar vertebrae, acetabula, and sacra, indicating the presence of metastatic disease (Figure 2). Subsequently, an incisional biopsy of the penis was performed, and the pathological findings microscopically revealed small undifferentiated round cell tumors with monomorphic cytomorphology, round-oval nuclei (Figure 3). Immunohistochemical stain-identified tumor cells are a positive expression of CD99, Ki-67 (40%), and a focally positive expression of FLI-1 but negative to neuron-specific enolase, desmin, and myogenin. The fluorescence *in situ* hybridization (FISH) identified the translocation of EWS gene, consequently confirming the diagnosis of Ewing sarcoma (Figure 3). Further histopathological investigation of femoral lesions was performed, and the punch-biopsy specimen showed malignant small round cells consistent with the microscopic presence of Ewing sarcoma. Based on the patient's medical history, as well as our radiological and pathological findings, the diagnosis of primary EES of the penis with multiple metastases was established. Given the patient's advanced metastatic disease, systemic chemotherapy consisting of vincristine, doxorubicin, and ifosfamide was initiated. However, after the first course of chemotherapy, the mass of the penis did not obtain stabilization but instead grew progressively with surface ulceration. The patient also suffered a persistent fever, and bacterial culture results then showed a mixed multidrug-resistant bacterial infection, including pseudomonas aeruginosa, klebsiella pneumoniae, and Escherichia coli. In the subsequent 2 months, the symptoms of the patient's infections became worse, associated with hypoalbuminemia, severe anemia, multiple-organ dysfunction, and tumor progression. Unfortunately, despite receiving antibiotics and maximal supportive therapy, the patient died from sepsis and complications from lung metastasis in the intensive care unit 2 months later.

## Discussion

As a subtype of Ewing sarcoma, EES is far less frequent than skeletal Ewing sarcoma (SES). EES may develop in soft tissues at virtually any anatomical location, but the main sites of involvement are the paravertebral region, the trunk, and the extremities (4, 6, 7, 10–13). However, primitive localization in the penis is extremely rare; only a few relevant cases have been reported in the previous literature (8, 9, 14, 15). Due to the lack of specific clinical, radiological features and the rarity of the condition, it can easily be misdiagnosed by surgeons and radiologists. As in the presented case, the patient was initially misdiagnosed with priapitis and treated with antibiotics. The most common clinical manifestation of EES is a rapidly growing mass with or without local pain (16). Moreover, the radiologic characteristics of EES are non-specific as well; it is frequently presented as a well-limited soft tissue mass with or without adjacent organ invasion on MRI that can be confused with benign lesions (16).

Currently, diagnosis of EES largely depends on pathological and immunohistochemical findings. Microscopically, EES presents distinctively monomorphic round blue cells, which are characterized by round, oval sheet cells with primitive nuclei and clear cytoplasm. On an immunohistochemical stain, CD99 and friend leukemia integration 1 transcription factor (FLI1) represent the major diagnostic markers. Diffuse membranous positive expression of CD99, a cell-surface glycoprotein, is evident in nearly all Ewing sarcomas (17), whereas CD99 expression is not exclusively specific for Ewing sarcoma and its immunopositivity also occurs in a large group of normal tissues or other round cell sarcomas (18). FLI1 is more specific for Ewing sarcoma than CD99, but positive FLI1 can be found also in lymphoblastic leukemias, lymphomas, and several soft-tissue sarcomas (19). Additionally, its sensitivity is limited by the occurrence of variant translocations not involving FLI1 (19). More than 85% of patients with Ewing sarcoma harbor the reciprocal translocation between the EWS and FLI1 genes, t(11;22) (q24;q12), resulting in the EWSR1-FLI1 fusion transcript (20). Molecular pathology by FISH analysis or reverse transcription PCR plays a crucial role in diagnosing Ewing sarcoma, particularly when microscopy and immunohistochemistry are not specific. A differential diagnosis needs to exclude other small round blue cell tumors that involve bone and soft tissue, including lymphoma, small cell osteosarcoma, mesenchymal chondrosarcoma, undifferentiated neuroblastoma, synovial sarcoma, desmoplastic small round cell tumors, and rhabdomyosarcoma. In the present case, immunohistochemistry results showed that CD99 was positive, FLI-1 was focally positive, and FISH evaluation confirmed the EWS gene translocation. As a result, the diagnosis of Ewing sarcoma was established.

However, the pathological finding of both lesions in the femur and penis was consistent with Ewing sarcoma, making it difficult to exclude the possibility of penile metastasis from the skeletal system. Presently, there are no conclusive criteria or guidelines regarding identifying the primary location of Ewing sarcoma in both the bone and soft tissue concurrently. In previous literature, Cash et al. and Applebaum et al. have stated that any tumor with any degree of bone involvement was considered a primary bone tumor, but they did not mention the condition that extra-skeletal diseases extended to the skeleton (21, 22). Huh et al. and Somarouthu et al. have found that 8.8–31% of patients with EES had metastatic osseous lesions at the initial diagnosis, indicating that bone involvement is insufficient to identify the origination of Ewing sarcoma (23, 24). On contrast-enhanced CT or MRI, Ewing sarcoma was typically characterized as a large mass with local invasion or a mass effect on adjacent organs (23, 25). Patients' EES tended to arise in axial locations with smaller tumors (size <8 cm) as compared with patients with SES (21). Similar to the PET/CT findings of classic Ewing sarcoma, EES presents uptake of ^18^f-fluorodeoxyglucose with an average maximum SUV value ranging from 3 to 11 (24). PET/CT has also been identified as a useful modality for detecting distant metastases, especially when bone metastasis remains occult on CT (24). In the present case, the MRI images demonstrated a bulky, soft-tissue mass with the invasion of adjacent tissue in the dorsal side of the penis, while the lesion of the left femur was limited to the medullary cavity without extraosseous extension. The pelvic CT displayed an aggressive lytic lesion involving the left femoral neck and trochanteric region with cortical destruction and limited periosteal reaction. These radiological features of the femoral lesion, including limited periosteal reaction and soft tissue component, largely support the diagnosis of bone metastasis (26). The PET/CT further revealed multiple uptake lesions in the ribs, lumbar vertebrae, acetabula, and sacra, suggesting the widespread metastasis of the patient's skeleton. Moreover, bone metastases derived from soft tissue sarcoma are much more frequent than soft tissue metastases from bone sarcoma. Consequently, based on the imaging and pathological findings, the diagnosis of primary EES in the penis with multiple metastases was established.

Although, to make the therapeutic decision, the skeletal or extra-skeletal origin is not considered, EES shows different clinical features and outcomes from SES. The overall survival rate of patients with localized EES is comparable with that of patients with SES, but EES patients with metastasis suffered a worse survival rate (16, 22, 27). Notably, patients with EES tend to have a higher incidence of distant metastases than SES patients. It has been reported that 30–40% of EES patients had distant metastatic disease at the time of diagnosis (24, 28). The lungs are the most common site of metastasis, followed by the lymph nodes and bones (24, 29). Besides, the local recurrent rate of EES is also higher than SES. The potential reason is attributed to the larger proportion of axial tumors in EES, a site that may be less amenable to complete resection with negative margins (21). Additionally, metastases of the regional lymph nodes may result in an incomplete resection during surgery that increases the risk of recurrence (23, 30).

The National Comprehensive Cancer Network (NCCN, Version 1.2020) has stated that any member of the Ewing tumor family can be treated according to the same protocol as SES, including systematic chemotherapy and local treatment (31). Current regimens of chemotherapy include vincristine, doxorubicin, and cyclophosphamide, alternating with ifosfamide and etoposide. Neoadjuvant chemotherapy is recommended prior to local therapy, and adjuvant chemotherapy is necessary for all patients regardless of margin status. Extensive resection is still the mainstay of local control for patients with resectable diseases. Of note, surgical margin plays a more important role in EES compared to SES since complete resection of EES has been proposed as a predictor of favorable survival (21, 22). Therefore, resection with a tumor-free surgical margin should be considered the primary surgery goal for a better clinical outcome. For patients with EES at locations that are not amenable for complete resection, such as the spine or pelvis, postoperative radiotherapy is recommended to minimize the risk of local recurrence (32, 33).

## Conclusion

EES is a rare subtype of Ewing sarcoma that originates from extra-skeletal sites. Due to the lack of specific clinical manifestation and radiological features, the definitive diagnosis of EES is mainly based on pathological and molecular assessment. Patients with EES tend to have a high incidence of distant metastasis and local recurrence, which lead to a worse prognosis. Therefore, we emphasize that clinicians should recognize this entity from other soft tissue sarcomas with rapid progression because early diagnosis and timely treatment of EES are pivotal for a favorable oncological outcome.

## Data Availability Statement

The original contributions generated for this study are included in the article/supplementary materials, further inquiries can be directed to the corresponding author/s.

## Ethics Statement

This study was approved by the West China Hospital's ethics committee, Sichuan University (Chengdu, People's Republic of China), and was permitted to be published. Written informed consent to have the case details and accompanying images published was obtained from the patient's parents. All clinical investigations were conducted following the principles expressed in the Declaration of Helsinki.

## Author Contributions

All authors contributed to data collecting, drafting, or revising the article, giving final approval of the version to be published, and agreeing to be accountable for all aspects of the work.

## Conflict of Interest

The authors declare that the research was conducted in the absence of any commercial or financial relationships that could be construed as a potential conflict of interest.
